# Augmentation of Omicron BA.1 pathogenicity in hamsters using intratracheal inoculation

**DOI:** 10.1038/s44298-023-00012-2

**Published:** 2024-01-16

**Authors:** Julia R. Port, Claude Kwe Yinda, Claire Ruckel, Jonathan E. Schulz, Brian J. Smith, Carl I. Shaia, Vincent J. Munster

**Affiliations:** 1 Laboratory of Virology, Division of Intramural Research, National Institute of Allergy and Infectious Diseases, National Institutes of Health, Hamilton, MT USA; 2 Rocky Mountain Veterinary Branch, Division of Intramural Research, National Institute of Allergy and Infectious Diseases, National Institutes of Health, Hamilton, MT USA

**Keywords:** Microbiology, Virology, SARS-CoV-2, Viral vectors, Virus-host interactions

## Abstract

The Omicron BA.1 variant of SARS-CoV-2 displays an attenuated phenotype in the Syrian hamster after intranasal inoculation. This is characterized by reduced viral replication and lung pathology in the lower respiratory tract. Here, we report that intratracheal inoculation with Omicron BA.1 recovers the lower respiratory tract replication and pathogenicity as observed with other lineages.

## Introduction

Omicron BA.1 displays an attenuated pathogenic phenotype in several animal models; virus replication in the lower respiratory tract is reduced both in rodents^1,2^ and non-human primates^3–5^. Omicron BA.1 also displays a reduced transmission efficiency compared to Alpha and Delta variants of concern (VoC) in hamsters^6^. Here we investigated whether intratracheal inoculation in hamsters, which directly deposits the virus to the lower respiratory tract, would overcome this attenuation.

**Fig. 1:**
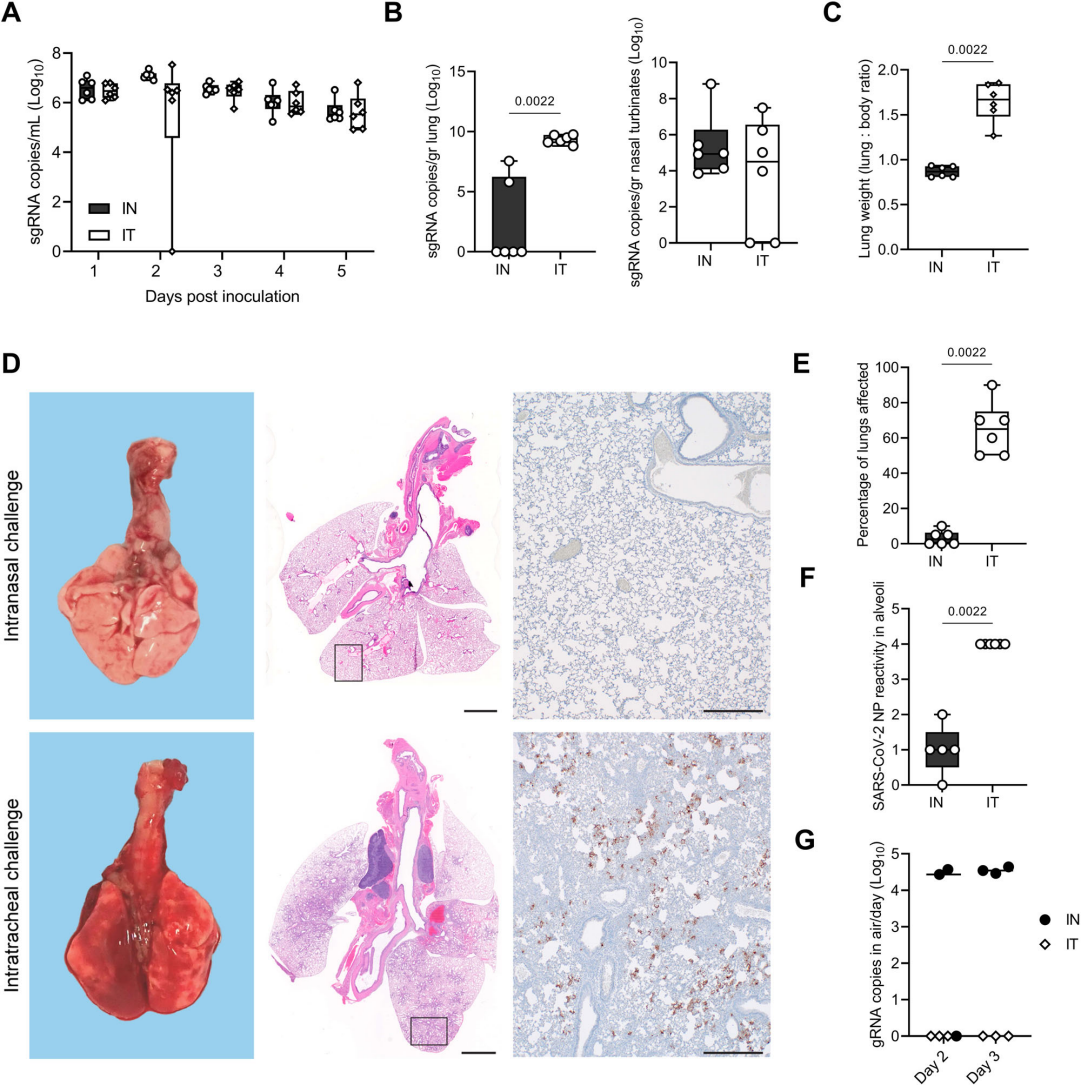
Syrian hamsters were inoculated with Omicron BA.1 through the intranasal (IN) or intratracheal (IT) route (group size *N* = 6). Shedding and virus titers in tissues were compared. **A** Viral load as measured by subgenomic (sg) RNA in oropharyngeal swabs collected at 1–5 days post inoculation. Whisker-plots depicting median, min and max values, and individual values, *N* = 6. **B** sgRNA in lungs and nasal turbinates on day 5. Whisker-plots depicting median, min, and max values, and individual values. Mann–Whitney test, *N* = 6. **C** Lung weights (lung : body ratio). Whisker-plots depicting median, min and max values, and individual values, Mann–Whitney test, *N* = 6. **D** Gross pathology of lungs on IN (top) and IT (bottom) inoculated animals on day 5 (left), histopathology (HE, middle), and immunohistochemistry against SARS-CoV-2 NP antigen (IHC, ×200, right) **E** Percentage of lungs affected. **F** Quantitative analysis of the NP reactivity. Whisker-plots depicting median, min, and max values, and individual values. Mann-Whitney test, *N* = 6. **G** For each airborne transmission, cage air was sampled in 24 h intervals. Measurement of each individual cage is shown for genomic RNA. black = IN, white = IT. *P* values stated were significant (<0.05).

In the hamster model, it is proposed that the loss of TMPRSSII cleavage affinity^7^ and reduced entry of the Omicron BA.1 leads to a reduction in virus replication and pathogenicity in the lower respiratory tract; while the virus may replicate in the upper respiratory tract if an infection is established after low volume intranasal (IN) inoculation, it cannot cross into the lower respiratory tract unaided. However, replication and shedding from the upper respiratory tract remain comparable to other VoCs. This could suggest that the route of administration is a key factor to overcome the observed attenuation. We compared the intratracheal (IT) inoculation with the IN route for B.1.1.529. BA.1 at a high dose (10,000 TCID_50_). The shedding profile and magnitude for both inoculation routes were identical (Fig. 1A). While we did not observe the weight gain seen in healthy animals of this age (SI Fig. 1), no significant weight loss was observed up until day 5. However, after IT inoculation, significantly higher viral loads were observed in the lungs (median subgenomic (sg) RNA copies/gram (Log_10_): IN = 0, IT = 9.358, *p* = 0.0022, Mann–Whitney test, *N* = 6). Whereas viral loads in the nasal turbinates were similar (Fig. 1B). This was accompanied by a significant increase in lung pathology in the IT inoculated animals (Fig. 1C), measured by increased lung:body weight ratio at day 5 in the IT group (lung:body (%) = 0.8662 (IN)/1.669 (IT), *p* = 0.0022, Mann–Whitney test, *N* = 6) and observable lung lesions by gross pathology (50–90% of lobes affected) (Fig. 1D). Histopathological lesions and SARS-CoV-2 antigen (*p* = 0.0022, Mann–Whitney test, *N* = 6) in the alveoli were consistent with what has previously been described for other SARS-CoV-2 VoCs^8,9^ (Fig. 1E, F, Table 1). After a low dose of inoculation with the same virus (100 TCID_50_), no weight loss was observed, lung weight on day 5 remained lower compared to both high dose inoculation routes, and sgRNA in nasal turbinates was comparable to the high dose inoculations but was reduced in the lungs; the impact of the inoculation route at a low dose of Omicron BA.1 inoculation was less impactful as compared to the high dose inoculation.

**Table 1:** Pathological assessment of IN and IT inoculated Syrian hamsters on day 5.

	Intranasal inoculation						Intratracheal inoculation					
Lesions visible grossly	no	no	no	yes	no	no	yes	yes	yes	yes	yes	yes
Percentage of total area affected	0	<5	0	10	5	0	50	90	70	50	70	60
Alveolar inflammation or exudate	0	0	1	2	1	1	4	4	4	4	4	4
Bronchiolar epithelial cell inflammation/necrosis	0	1	1	0	0	0	1	0	0	0	0	0
Endotheleitis, vasculitis	no	no	no	yes	no	no	yes	yes	yes	yes	yes	Yes
Hemorrhage, fibrin, and or edema	0	0	0	0	0	0	0	3	3	0	2	0
SARS-CoV-2 IHC bronchioles	0	1	1	1	1	1	0	1	0	0	0	0
SARS-CoV-2 IHC alveoli	0	1	1	2	1	1	4	4	4	4	4	4

Pathological scoring: 0 = none, 1 = rare to mild, 2 = moderate, 3 =severe, 4= marked, 5= severe. Immunohistochemistry scoring (IHC): 0 = none, 1 = rare/few, 2 = scattered, 3 = moderate, 4 =numerous, 5 = diffuse. Slides were analyzed by a board-certified veterinary pathologist.

We then assessed if the change in tropism by inoculation route translated into differences in virus transmission (high dose, 10,000 TCID_50_). Sentinels were exposed for 48 h starting on day 1 (1:1 donor:sentinel ratio) by contact or by airborne exposure to IN or IT-infected donors. In the IN group, transmission occurred in 3 out of 3 contact sentinels and 1 out of 3 air sentinel determined by detection of sgRNA and seroconversion (Table 1, previously shown in ref. ^6^). In the IT group, transmission occurred in 1 out of 3 in the contact sentinels and no airborne transmission was observed (Table 2). SARS-CoV-2 gRNA could only be recovered in air samples on days 1 and 2 of exposure if the donor animal was IN inoculated, but not IT inoculated (Fig. 1G).

**Table 2:** Contact and airborne transmission efficiency (virus shedding via subgenomic (sg) RNA^♯^ measurement) after intranasal and intratracheal inoculation.

	Intranasal inoculation						Intratracheal inoculation					
Groups	Contact transmission			Airborne transmission			Contact transmission			Airborne transmission		
Sentinel animal	1	2	3	1	2	3	1	2	3	1	2	3
sgRNA shedding day 3	0	5.1	5.1	0	0	5.6	6.3	0	6.7	0	0	0
sgRNA shedding day 5	6.7	5.9	5.7	0	0	6.1	5.2	0	0	0	0	0
Seroconversion	+	+	+	−	−	+	+	−	+	−	−	−

# = sgRNA shedding in copies/mL (Log10), + = positive, − = negative. Seroconversion of sentinels was measured by anti-spike SARS-CoV-2 IgG ELISA and values are the average of two replicates, diluted 1:100. Cutoff = OD of 0.07 for positivity.

Intratracheal inoculation with Omicron BA.1 at a high but not a low infection dose displays a lower respiratory tract replication and lung pathology phenotype similar to that seen after intranasal inoculation with the other VoCs in the Syrian hamster^10,11^. High-volume IN inoculations have also reported an augmentation of pathology^12^, most likely through direct inhalation of inoculum into the lungs. This suggests that the initial site of virus deposition can overcome the attenuated disease phenotype observed with Omicron BA.1 in hamsters^7^. Contact transmission efficiency with Omicron BA.1 was not markedly reduced after intratracheal inoculation, and no airborne transmission was observed in contrast to IN transmission pairs. The lack of airborne transmission in the IT groups combined with the absence of viral RNA in air samples, suggests that replication in the upper respiratory tract is required for airborne transmission but not for contact transmission. Importantly, these findings also suggest that using the IT inoculation route, which allows controlled and exact application of inoculum to the lower respiratory tract, the young adult Syrian hamster shows robust lung pathology, remaining an invaluable model for the initial pre-clinical development of prophylactic and therapeutic countermeasures against SARS-CoV-2.

## Methods

### Ethics statement

All animal experiments were conducted in an AAALAC International-accredited facility and were approved by the Rocky Mountain Laboratories Institutional Care and Use Committee following the guidelines put forth in the Guide for the Care and Use of Laboratory Animals 8th edition, the Animal Welfare Act, United States Department of Agriculture and the United States Public Health Service Policy on the Humane Care and Use of Laboratory Animals. Protocol number 2021-034-E. Work with infectious SARS-CoV-2 virus strains under BSL3 conditions was approved by the Institutional Biosafety Committee (IBC). For the removal of specimens from high containment areas, virus inactivation of all samples was performed according to IBC-approved standard operating procedures^13^.

### Cells and viruses

Virus propagation was performed in VeroE6 cells in DMEM supplemented with 2% fetal bovine serum, 1 mM L-glutamine, 50 U/mL penicillin, and 50 μg/mL streptomycin (DMEM2). VeroE6 cells were maintained in DMEM supplemented with 10% fetal bovine serum, 1 mM L-glutamine, 50 U/mL penicillin, and 50 μg/ml streptomycin. Mycoplasma testing was performed at regular intervals. No mycoplasma or contaminants were detected. All virus stocks were sequenced, and no SNPs compared to the patient sample sequence were detected.

### Comparison between intranasal and intratracheal inoculation

Four-to-six-week-old male Syrian hamsters (ENVIGO) were randomly assigned to two groups, intratracheal and intranasal inoculation, and inoculated with 1 × 10^4^ TCID_50_ Omicron BA.1 (B.1.1.529; hCoV-19/USA/GA-EHC-2811C/2021) in a volume of 40 µL (IN) or 100 µL (IT) (*N* = 6). For intranasal challenge, hamsters were inoculated intranasally using 20 µL per nostril using a pipette. For intratracheal challenge, hamsters were restrained on a hang board, the tongue was retracted with tissue-friendly forceps, the trachea visualized with a speculum and light source, and a 21–25 gauge flexible catheter tip attached to 1 mL syringe was used. Animals were then individually housed, and swabbed daily in the oropharyngeal cavity, and lungs and nasal turbinates collected at day 5. On day 1, each animal was either co-housed with a naïve sentinel (contact, *N* = 3) or placed into the upstream cage of a short-distance aerosol transmission cage (16.5 cm), and one sentinel placed adjacent (air, *N* = 3). Animals were exposed at a 1:1 ratio, for 48 h. Air was sampled in 24 h intervals for the air transmission set-ups as described previously^14,15^. Sentinels were swabbed on days 3 and 5 post exposure, and serum was collected on day 14.

### Low dose inoculation

Four-to-six-week-old male Syrian hamsters (ENVIGO) were randomly assigned to two groups, intratracheal and intranasal inoculation, and inoculated with 1 × 10^2^ TCID_50_ Omicron BA.1 in a volume of 40 µL (intranasal) or 100 µL (intratracheal) (*N* = 4). Lungs and nasal turbinates were collected at day 5.

### Viral RNA detection

140 µL of swab media was utilized for RNA extraction using the QIAamp Viral RNA Kit (Qiagen) using QIAcube HT automated system (Qiagen) according to the manufacturer’s instructions with an elution volume of 150 µL. For tissues, RNA was isolated using the RNeasy Mini kit (Qiagen) according to the manufacturer’s instructions and eluted in 60 µL. Subgenomic (sg) and genomic (g) viral RNA were detected by qRT-PCR^16^. RNA was tested with TaqMan™ Fast Virus One-Step Master Mix (Applied Biosystems) using QuantStudio 3 Flex Real-Time PCR System (Applied Biosystems). SARS-CoV-2 standards with known copy numbers were used to construct a standard curve and calculate copy numbers/mL or copy numbers/g. The detection limit for the assay was 10 copies/reaction, and samples below this limit were considered negative.

### Virus titration

Viable virus in tissue samples was determined as previously described^17^. In brief, lung tissue samples were weighed, and then homogenized, in 1 mL of DMEM (2% FBS). Swabs were used undiluted. VeroE6 cells were inoculated with tenfold serial dilutions of homogenate, incubated 1 h at 37 °C, and the first two dilutions were washed twice with 2% DMEM. For swab samples, cells were inoculated with tenfold serial dilutions, and no wash was performed. After 6 days, cells were scored for cytopathic effect. TCID_50_/mL was calculated by the method of Spearman–Karber. A plaque assay was used to determine titers in air samples. VeroE6 cells were inoculated with 200 µL/well (48-well plate) of undiluted samples, with no wash performed. Plates were spun for 1 h at room temperature at 1000 rpm. 800 µL of CMC (500 mL MEM (Cat#10370, Gibco, must contain NEAA), 5 mL PenStrep, 7.5 g carboxymethylcellulose (CMC, Cat# C4888, Sigma, sterilized in an autoclave) overlay medium was added to each well and plates incubated for 6 days at 37 °C. Plates were fixed with 10% formalin overnight, then rinsed and stained with 1% crystal violet for 10 min. Plaques were counted.

### ELISA

Serum samples were analyzed as previously described^18^. In brief, maxisorp plates (Nunc) were coated with 50 ng spike protein (generated in-house) per well. Plates were incubated overnight at 4 °C. Plates were blocked with casein in phosphate-buffered saline (ThermoFisher) for 1 h at room temperature. Serum was diluted twofold in blocking buffer, and samples (duplicate) were incubated for 1 h at room temperature. Secondary goat anti-hamster IgG Fc (horseradish peroxidase (HRP)-conjugated, Abcam) spike-specific antibodies were used for detection and visualized with KPL TMB 2-component peroxidase substrate kit (SeraCare, 5120-0047). The reaction was stopped with KPL stop solution (Seracare) and plates were read at 450 nm. The threshold for positivity was calculated as the average plus 3× the standard deviation of negative control hamster sera.

### Histopathology

Necropsies and tissue sampling were performed according to IBC-approved protocols. Tissues were fixed for a minimum of 7 days in 10% neutral buffered formalin with 2 changes. Tissues were placed in cassettes and processed with a Sakura VIP-6 Tissue Tek, on a 12-hour automated schedule, using a graded series of ethanol, xylene, and PureAffin. Prior to staining, embedded tissues were sectioned at 5 µm and dried overnight at 42 °C. Using GenScript U864YFA140-4/CB2093 NP-1 (1:1000) specific anti-CoV immunoreactivity was detected using the Vector Laboratories ImPress VR anti-rabbit IgG polymer (# MP-6401) as secondary antibody. The tissues were then processed using the Discovery Ultra automated processor (Ventana Medical Systems) with a ChromoMap DAB kit Roche Tissue Diagnostics (#760-159). Anti-CD3 immunoreactivity was detected utilizing a primary antibody from Roche Tissue Diagnostics predilute (#790-4341), secondary antibody from Vector Laboratories ImPress VR anti-rabbit IgG polymer (# MP-6401), and visualized using the ChromoMap DAB kit from Roche Tissue Diagnostics (#760-159). Anti-PAX5 immunoreactivity was detected utilizing a primary antibody from Novus Biologicals at 1:500 (#NBP2-38790), secondary antibody from Vector Laboratories ImPress VR anti-rabbit IgG polymer (# MP-6401), and visualized using the ChromoMap DAB kit from Roche Tissue Diagnostics (#760-159).

### Statistics

Animals were randomly assigned to the experimental groups; investigators were not blinded to allocation during the experiments but were blinded during outcome assessment. Data distribution was assumed to be non-normal and non-parametric tests were applied where appropriate. No data or animals were excluded from the analysis. Significance tests were performed as indicated where appropriate using Prism 9 (GraphPad Software). Statistical significance levels were determined as follows: NS, *P* > 0.05; *P ≤ 0.05; **P ≤ 0.01; ***P ≤ 0.001; ****P ≤ 0.0001.

## Data Availability

All data are available in the main text, the supplementary materials, and on FigShare: 10.6084/m9.figshare.24878253.v1.

## Supporting Information

Supporting information
