# Supplementary material for: Augmentation of Omicron BA.1 pathogenicity in hamsters using intratracheal inoculation
Source: Npj Viruses. 2024 Jan 16;2:3. doi: 10.1038/s44298-023-00012-2 (PMC11702663; doi:10.1038/s44298-023-00012-2)
Supplement: Supplementary file 1 — Supporting information [file 44298_2023_12_MOESM1_ESM.pdf]

Supporting Information for

Augmentation of Omicron BA.1 pathogenicity in hamsters using intratracheal inoculation

Julia R. Port*#[1], Claude Kwe Yinda[1], Claire Ruckel[1], Jonathan E. Schulz[1], Brian J. Smith[2], Carl. I. Shaia[2], Vincent J. Munster§[1]

1. Laboratory of Virology, Division of Intramural Research, National Institute of Allergy and Infectious Diseases, National Institutes of Health, Hamilton, MT, USA
2.. Rocky Mountain Veterinary Branch, Division of Intramural Research, National Institute of Allergy and Infectious Diseases, National Institutes of Health, Hamilton, MT, USA*

Julia R. Port
Email: julia.port@nih.gov

This PDF file includes:

Supplementary Figure 1

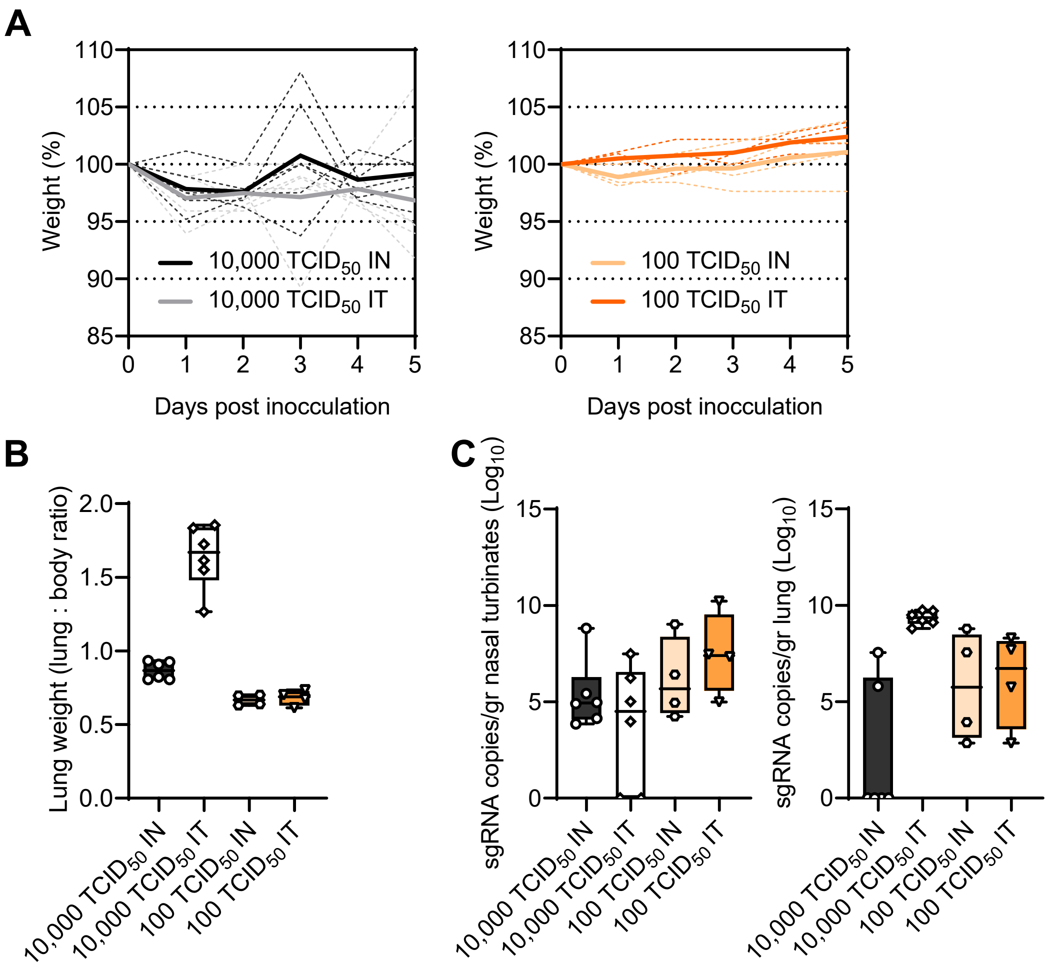

Supplementary Figure 1. Syrian hamsters were inoculated with Omicron BA.1 through the intranasal (IN) or intratracheal (IT) route with either 10,000 TCID$_{50}$ (group size N = 6) or 100 TCID$_{50}$ (group size N =4). **A.** Weights. **B**. Lung weights (lung : body ratio). Whisker-plots depicting median, min and max values, and individual values **C.** subgenomic (sg) RNA in lungs and nasal turbinates on day 5. Whisker-plots depicting median, min and max values, and individual values. Data for the higher inoculation dose is shown again for visualization purposes. black = 10,000 TCID$_{50}$ IN, white/grey = 10,000 TCID$_{50}$ IT, salmon = 100 TCID$_{50}$ IN, orange = 100 TCID$_{50}$ IT.
